# Aneuploidy in neuroblastoma tumors is not associated with inactivating point mutations in the *STAG2* gene

**DOI:** 10.1186/1471-2350-14-102

**Published:** 2013-10-02

**Authors:** Anna Djos, Susanne Fransson, Per Kogner, Tommy Martinsson

**Affiliations:** 1 Department of Clinical Genetics, Institute of Biomedicine, University of Gothenburg, Sahlgrenska University Hospital, SE-413 45 Gothenburg, Sweden; 2 Childhood Cancer Research Unit, Department of Woman and Child Health, Karolinska Institute, Karolinska University Hospital, SE-17176 Stockholm, Sweden

**Keywords:** Neuroblastoma, STAG2, Aneuploidy, Numerical aberrations, Chromosomal instability

## Abstract

**Background:**

Chromosomal instability is a hallmark of human cancer caused by errors in mitotic control and chromosome segregation. *STAG2* encodes a subunit of the cohesion complex that participates in mitotic chromatid separation and was recently found to show low expression and inactivating mutations in Ewing’s sarcoma, melanoma and glioblastoma.

In the childhood tumor neuroblastoma (NB) segmental chromosomal alterations are associated with poor prognosis whereas tumors displaying whole chromosome gains and losses have a much better prognosis.

**Method:**

As the genetic contribution to aneuploidy is unknown in NB, we investigated the presence of *STAG2* mutations through sequence analysis of all 33 coding exons in 37 primary NB tumors.

**Results and conclusion:**

As no STAG2 mutation was detected in this study, we conclude that inactivating mutation of *STAG2* is not likely causative to neuroblastoma aneuploidy.

## Background

Chromosome instability leading to numerical and segmental alteration is a hallmark of human cancer genomes, contributing to malignant transformation. Whereas segmental rearrangements directly can promote cancer through activating oncogenes and inactivating tumors suppressor genes, aneuploidy could provide a tumor driving function through dosage-dependent mechanisms [1]. Aneuploidy is caused by inaccurate chromosome segregation, possibly due to genetic alteration of various participants of the segregation or mitotic checkpoint machinery. Recently, Solomon et al., showed the presence of inactivating mutations in the *STAG2* gene located at Xq25 in primary tumors and cancer cell lines originating from glioblastoma, melanoma and Ewing’s sarcoma. The *STAG2* mutations reported by Solomon et al., represented an array of different alterations (missense-, nonsense-, splice site mutations as well as intragenic deletions), mainly concentrated to hotspot regions corresponding to exon 9, 11, 12 and 20 [2]. *STAG2* encodes a subunit of the multimeric cohesion complex required in regulating sister chromatid separation during mitosis [3-5]. Inactivation of *STAG2* in cell lines with stable karyotype resulted in increased number of abnormal mitotic events (e.g. lagging chromosomes and anaphase bridges) ultimately leading to increased aneuploidy rate while mutational correction of endogenous *STAG2* mutations in aneuploid cell lines resulted in enhanced chromosomal stability. Further, loss of STAG2 protein was observed in several human cancer cell lines, primary tumors and xenografts of different origin [2].

Neuroblastoma (NB), a childhood cancer of the postganglionic nervous system show broad biological and clinical heterogeneity, ranging from highly aggressive tumors with fatal outcome to tumors with spontaneous regression. Advanced disease and poor prognosis in NB patients is commonly associated with either *MYCN* amplification or 11q-deletion, representing two separate NB subgroups with different genomic profiles. Whereas *MYCN* amplified tumors commonly have few segmental or numerical aberrations besides 17q-gain and 1p-deletions, 11q-deleted NB generally contains many more segmental alterations. In contrast, NB tumors with numerical aberrations (i.e. only whole chromosome gains and/or losses) without other segmental alterations instead have excellent outcome [6] and the underlying mechanisms causing aneuploidy in NB tumors are yet unknown.

As the findings of Solomon et al [2] indicate that loss of functional *STAG2* protein could contribute to increased chromosomal instability we wanted to investigate the presence of inactivating mutations in *STAG2* in prognostically favorable NB with common aneuploidy pattern and prognostically unfavorable NB with high degree of segmental alteration.

## Methods

### Cell material

Genomic DNA of 37 primary NB tumors and cell lines was extracted with DNeasy blood and tissue kit (Qiagen, Hilden Germany) according to the manufacturer’s instructions. Mutation analysis through Sanger sequencing of the 33 coding exons (3-35) of *STAG2* was performed on 18 favorable NB tumor samples (stage 1, 2 and 4S), whereas additional 19 tumors of different risk groups were subjects for whole exome sequencing whereof one was also evaluated through Sanger sequencing (Table 1). In addition, mutation analysis with Sanger sequencing was performed on eleven NB cell lines (Table 2) SK-N-AS, SK-N-BE(2), SK-N-F1, SH-SY-5Y, SK-N-SH, SK-N-DZ, NB69, Kelly, IMR32, LS and NGP) for exons 3, 9, 11, 12, 20, 23 and 25. Informed consent was obtained from the parents of the patients. Ethical permission was granted by the ethics committee (Karolinska Institutet and Karolinska University Hospital, registration number 03-736 and 09-1369).

**Table 1:** Clinical and biological data of the tumors

**Sample**	**INSS**	**INRG**	**Gender**	**1p-del**	**MNA**	**11q-del**	**17q-gain**	Genomic profile*	**5-year OS**	**Sanger**	**NGS**
3E2	4	M	Ma	neg	neg	pos	pos	11q-del	DOD		+
4E1	4	M	F	neg	neg	pos	pos	11q-del	DOD		+
5E1	2A	L	F	neg	neg	neg	neg	Num only	NED	+	
6E9	3	L	Ma	pos	neg	pos	pos	11q-del	DOD		+
8E4	1	L	F	neg	neg	neg	neg	Num only	NED	+	
8E5	3	L	F	neg	neg	neg	neg	Num only	NED	+	
11E1	4	M	F	neg	neg	pos	pos	11q-del	NED		+
11E4	4	M	F	neg	neg	pos	pos	11q-del	DOD		+
11E8	1	L	Ma	neg	neg	neg	neg	Num only	NED	+	+
12E5	4S	MS	Ma	neg	neg	neg	neg	Num only	NED	+	
13E7	4S	MS	F	neg	neg	neg	neg	Num only	NED	+	
15E7	3	L	Ma	neg	neg	neg	neg	Num only	DSC	+	
16E1	1	L	F	neg	neg	neg	neg	Num only	NED	+	
16E2	4S	L	Ma	neg	neg	neg	neg	Num only	NED	+	
17E5	4	M	F	neg	neg	neg	neg	Num only	DOD	+	
18E2	1	L	F	neg	neg	pos	pos	11q-del	NED		+
18E5	1	L	F	neg	neg	neg	neg	Num only	NED	+	
9R9	3	M	F	pos	neg	pos	pos	11q-del	DOD		+
10R6	2A	L	Ma	neg	neg	neg	neg	Num only	NED	+	
10R8	3	L	F	neg	neg	pos	neg	Num only	DOD		+
13R1	3	L	Ma	pos	pos	neg	pos	MNA	DOD		+
15R8	3	M	Ma	pos	neg	pos	pos	11q-del	NED		+
23R4	2	L	F	neg	neg	neg	neg	Num only	NED	+	
25R6	4	M	Ma	pos	pos	neg	pos	MNA	DOD		+
25R7	1	L	Ma	pos	neg	neg	neg	Other segm.	NED	+	
26R1	1	L	Ma	neg	neg	neg	neg	Num only	NED	+	
27R1	2A	L	F	neg	neg	neg	neg	Num only	NED	+	
27R7	2	L	Ma	neg	neg	neg	neg	Num only	NED	+	
28R8	4	M	Ma	neg	neg	pos	pos	11q-del	DOD		+
30R0	4	M	F	neg	neg	pos	neg	11q-del	NED		+
34R5	3	L	Ma	neg	neg	neg	neg	Num only	NED	+	
37R5	1	L	Ma	neg	neg	neg	neg	Num only	NED	+	
44R5	1	L	F	neg	neg	neg	neg	Num only	NED		+
46R6	4	M	Ma	neg	neg	pos	pos	11q-del	NED		+
47R4	4	M	F	neg	neg	pos	pos	11q-del	NED		+
49R1	4	M	Ma	neg	neg	pos	pos	11q-del	NED		+
52R2	1	L	Ma	neg	neg	neg	pos	17q-gain	NED		+

Abbreviations: *INSS* International Neuroblastoma Staging System [7], *INRG* International Neuroblastoma Risk Group [8], *M* metastatic, *L* localized, *Ma* male, *F* female, *neg* negative, *pos* positive, *MNA NMYC*-amplification, *NED* no evidence of disease, *DOD* dead of disease, *DSC* dead of surgical complications. *As defined by Carén et al [6].

**Table 2:** Characteristics of the cell lines

**Cell line**	**Gender**	**1p-del**	**MNA**	**11q-del**	**17q-gain**	**ChrX**
NGP	Ma	neg	pos	pos	pos	one
Kelly	F	neg	pos	pos	pos	one
LS	F	neg	pos	neg	pos	two
SH-SY-5Y	F	neg	neg	neg	pos	two
NB69	Ma	pos	neg	neg	pos	one
SK-N-FI	Ma	neg	neg	neg	pos	one
SK-N-AS	F	pos	neg	pos	pos	one
IMR32	Ma	pos	pos	neg	pos	one
SK-N-BE	Ma	neg	pos	neg	pos	one
SK-N-DZ	F	neg	pos	pos	neg	two
SK-N-SH	F	neg	neg	neg	pos	two

Abbreviations: *Ma* male, *F* female, *neg* negative, *pos* positive.

### High resolution SNP array analysis

**Figure 1:**
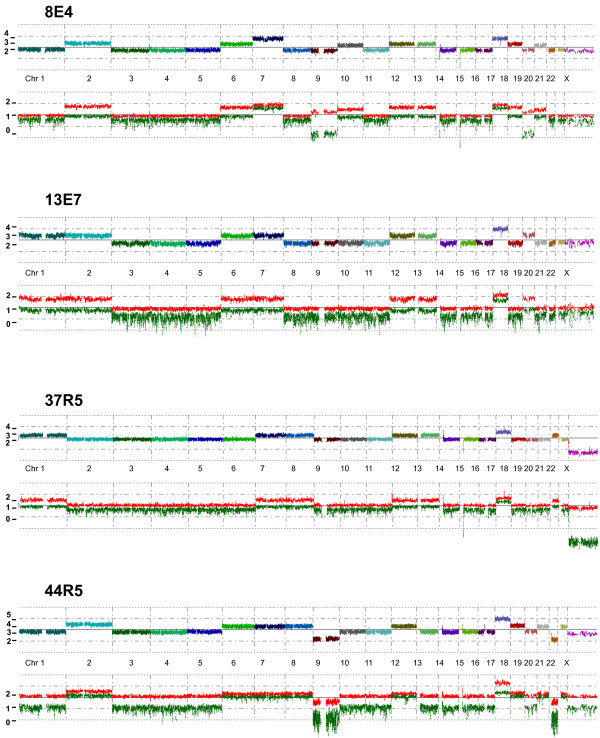
**SNP array genomic profiles of four representative Neuroblastoma tumors with numerical aberrations.** The total copy numbers of each chromosome are shown by the upper multicolored line and the red and green lower lines represent the strongest (red) and weakest (green) allele specific intensity.

Genomic profiling of the 37 primary NB tumors (Table 1, Figure 1) were performed with Genechip Human Mapping 250K NspI (Affymetrix Inc., Santa Clara, CA) according to the experimental procedure previously described [9].

### Sanger sequencing

Sanger sequencing was performed as earlier described [10] and primers were designed manually or with the use of ExonPrimer (http://ihg.gsf.de/ihg/ExonPrimer.html), primer sequences are available upon request. The sequence data were analyzed using Seqscape v2.5 (Applied Biosystems).

### Exome sequencing

Whole exome capturing was made with 5 μg gDNA using SureSelect Human All Exon Target Enrichment System 3.0 (Agilent Technologies, Santa Clara, CA) or TrueSelect exome enrichment kit (Illumina, San Diego, CA) before pair-end sequencing on Illumina HiScanSQ or HighSeq2000. Alignment to the hg19 reference genome (http://genome.ucsc.edu/) was performed using BWA [11], indel realignment with GATK [12] while functional variant annotation was made through ANNOVAR [13]. Visualisation for manual reviewing of alignments was performed using the DNAnexus software (DNAnexus Inc. Mountain View, CA) or the Integrative Genomic Viewer [14].

## Results

The coding sequence of the *STAG2* gene was screened with Sanger sequencing and targeted re-sequencing in a panel of 37 NB tumors and eleven NB cell lines (Tables 1 and 2). High-quality sequence of the *STAG2* gene was obtained for all 33 coding exons both from Sanger- and exome sequencing. Sequences with an average coverage of 51x (range 13-117x) were acquired from all coding exons in tumors analyzed by exome sequencing.

**Figure 2:**
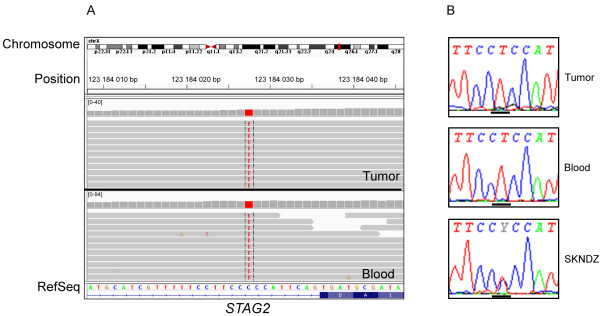
**A hemizygous/homozygous base substitution corresponding to SNP rs7063522 detected in intron 10. A)** Upper and lower panel show results from exon sequencing of tumor and blood DNA respectively from NB patient 11E8. **B)** Upper and middle panel show results from Sanger sequencing of the same NB patient’s tumor and blood DNA respectively, lower panel show result from Sanger sequencing of the NB cell line SK-N-DZ. Bars under the electropherograms show the position of the base substitution.

One of 37 primary tumors (11E8) and one of eleven NB cell lines (SK-N-DZ) showed a hemizygous and heterozygous base substitution respectively corresponding to C>T at mRNA position 1298-9 in intron 10 (Figure 2), similar to a finding earlier reported in a glioblastoma primary xenograft [2]. However, this variant corresponding to SNP rs7063522, was also present in normal DNA from patient’s blood (Figure 2). No exonic variants were detected in our set of 37 NB tumors and 11 NB cell lines, neither in the hot spot positions reported by Solomon et al., nor in any other coding region of the *STAG2* gene [2].

## Discussion

Aneuploidy results from missegregation of sister chromatids during mitotic cell division. In NB segmental chromosomal alterations are common in aggressive tumors whereas tumors with only whole chromosome gains and losses are prognostically more favorable [6]. However, the cause of the high aneuploidy rate in this favorable group of NB tumors is currently unknown. Aneuploidy is also frequently observed in other malignancies and recently *STAG2* was recognized as a tumor suppressor gene causing malfunctioning mitotic sister chromatid separation when inactivated. Inactivating mutations of *STAG2* have been found in glioblastoma, Ewing’s sarcoma and melanoma, possibly contributing to chromosome instability in these tumors [2] and this led us to investigate whether they might also be responsible for the high aneuploidy rate seen in favorable NB tumors.

In the present study, we sequenced all 33 coding exons of the *STAG2* gene in 11 NB cell lines and 37 primary NB tumors and no deviation from the human reference sequence could be detected. This is similar to other recent studies of the *STAG2* mutational hot spot region (corresponding to exon 9, 11, 12 and 20) in 90 acute leukemia samples [15] and 225 adult carcinomas of various origin (colorectal-, gastric-, breast-, prostate carcinoma and non-small lung cell cancer) [16]. Alteration of *STAG2* was not detected in either study, suggesting that *STAG2* inactivation is not due to inactivating gene mutations in these malignancies. Other gene silencing mechanisms might contribute as *STAG2* protein was lost in 23-30% of studied carcinomas [16].

According to the publicly accessible R2-microarray analysis and visualization platform (http://r2.amc.nl), *STAG2* expression in NB can be correlated with outcome in that lower *STAG2* expression is correlated to lower overall survival and relapse free survival. Other causes than *STAG2* mutations are likely to be involved in this, e.g. epigenetic events.

Furthermore, previous and subsequent analyzes of copy number variations performed by us in totally 360 primary NB tumors show that segmental deletions of the chromosomal region Xq25 harboring *STAG2* are not seen ([6] and unpublished data).

Taken together these data suggest that *STAG2* mutations are not likely responsible for the high aneuploidy rate in NB tumors with numerical aberrations. However, other genes participating in the mitotic checkpoint control may be responsible for the chromosomal missegregation in NB tumors with many numerical aberrations.

Three major next generation sequencing studies have together analyzed a large serie of NB tumors [17-19]. No *STAG2* mutations are reported and overall very few recurrent amino-acid changing mutations were found. Molenaar et al. [17], found few recurrent mutations except for the genes *ALK* and *TIAM1* and mutations in genes regulating neuritogenes. Cheung et al. [18], reported the finding of deletions and loss-of-function mutations in the *ATRX* gene and the association with advanced age at NB diagnosis. Furthermore, Sausen et al. [19], found mutations and deletions in the genes *ARID1A* and *ARID1B* which were associated with treatment failure and poor prognosis. Collectively more recurrent somatic mutation have been found in high risk NB patients compared to patients with stage 1, 2 and 4S tumors and in the published NGS data we cannot identify clear candidate genes that could be responsible for the high aneuploidy rate seen in favorable NB.

## Conclusion

Here we show that inactivating point mutations in the *STAG2* gene are not common in NB tumors and that aneuploidy seen in NB tumors is likely due to other participants of the mitotic checkpoint and chromosome segregation machinery.

## Competing interests

The authors declare they have no conflict of interests.

## Authors’ contributions

AD and SF performed experiments, analysis and wrote the paper. TM coordinated the project, critically reviewed the manuscript and provided biological and clinical information together with PK. All authors read and approved the final manuscript.

## Pre-publication history

The pre-publication history for this paper can be accessed here:

http://www.biomedcentral.com/1471-2350/14/102/prepub
